# Lesion detection in women breast’s dynamic contrast-enhanced magnetic resonance imaging using deep learning

**DOI:** 10.1038/s41598-023-48553-z

**Published:** 2023-12-18

**Authors:** Sudarshan Saikia, Tapas Si, Darpan Deb, Kangkana Bora, Saurav Mallik, Ujjwal Maulik, Zhongming Zhao

**Affiliations:** 1 Information Technology Department, Oil India Limited, Duliajan, Assam 786602 India; 2 AI Innovation Lab, Department of Computer Science & Engineering, University of Engineering & Management, Jaipur, GURUKUL, Jaipur, Rajasthan 303807 India; 3 Department of Computer Application, Christ University, Bengaluru, 560029 India; 4 Department of Computer Science and Information Technology, Cotton University, Guwahati, Assam 781001 India; 5 Department of Environmental Health, Harvard T. H. Chan School of Public Health, Boston, MA 02115 USA; 6 Department of Computer Science and Engineering, Jadavpur University, Kolkata, India; 7 Center for Precision Health, School of Biomedical Informatics, The University of Texas Health Science Center at Houston, Houston, TX 77030 USA

**Keywords:** Computational biology and bioinformatics, Diseases, Health care

## Abstract

Breast cancer is one of the most common cancers in women and the second foremost cause of cancer death in women after lung cancer. Recent technological advances in breast cancer treatment offer hope to millions of women in the world. Segmentation of the breast’s Dynamic Contrast-Enhanced Magnetic Resonance Imaging (DCE-MRI) is one of the necessary tasks in the diagnosis and detection of breast cancer. Currently, a popular deep learning model, U-Net is extensively used in biomedical image segmentation. This article aims to advance the state of the art and conduct a more in-depth analysis with a focus on the use of various U-Net models in lesion detection in women’s breast DCE-MRI. In this article, we perform an empirical study of the effectiveness and efficiency of U-Net and its derived deep learning models including ResUNet, Dense UNet, DUNet, Attention U-Net, UNet++, MultiResUNet, RAUNet, Inception U-Net and U-Net GAN for lesion detection in breast DCE-MRI. All the models are applied to the benchmarked 100 Sagittal T2-Weighted fat-suppressed DCE-MRI slices of 20 patients and their performance is compared. Also, a comparative study has been conducted with V-Net, W-Net, and DeepLabV3+. Non-parametric statistical test Wilcoxon Signed Rank Test is used to analyze the significance of the quantitative results. Furthermore, Multi-Criteria Decision Analysis (MCDA) is used to evaluate overall performance focused on accuracy, precision, sensitivity, F$_1$-score, specificity, Geometric-Mean, DSC, and false-positive rate. The RAUNet segmentation model achieved a high accuracy of 99.76%, sensitivity of 85.04%, precision of 90.21%, and Dice Similarity Coefficient (DSC) of 85.04% whereas ResNet achieved 99.62% accuracy, 62.26% sensitivity, 99.56% precision, and 72.86% DSC. ResUNet is found to be the most effective model based on MCDA. On the other hand, U-Net GAN takes the least computational time to perform the segmentation task. Both quantitative and qualitative results demonstrate that the ResNet model performs better than other models in segmenting the images and lesion detection, though computational time in achieving the objectives varies.

## Introduction

With the increase in the number of breast cancer cases, the field of medical science heavily depends upon its early detection for effective diagnosis and treatment in its early stages, but such a task has proven to be challenging. In the year 2020, over 2.3 million women were diagnosed with breast tumors and approximately 685,000 did not survive worldwide, as reported by the World Cancer Research Fund International and the World Health Organization. Breast cancer is the most frequent tumour found in women making it the most likely cancerous disease found in women. Breast cancer has slowly proven to be one out of the four most common cancers found in women. Globally, the cases have gone up by 20% and death rates have seen an increase of 14% since 2008^1^. Further study in this field of discipline can potentially make huge breakthroughs and may take the diagnosis and early detection process to a whole new dimension.

With the rapid advances in bio-technologies and medical technologies, the main progress has been made in cancer detection, diagnosis, and treatment. The best way of diagnosing breast cancer is through medical imaging tests. Medical imaging techniques used for diagnosis include ultrasound^2^, digital mammography^3^, magnetic resonance imaging^4^, microscopic slices^5^, and infrared thermograms^6^. As a method of assisting radiologists and doctors in recognizing issues, these modalities produce images that have decreased mortality rates by 30–70%^7^. Information technology is needed to speed up and increase the accuracy of diagnosis as well as provide a second opinion to the expert because picture interpretation is operator-dependent and requires ability^8^. Physicians can utilize computer-aided diagnosis (CAD) systems, which use computerized characteristics extraction and classification algorithms, to quickly identify anomalies.

It required a lot of effort to develop CAD systems based on developments in digital artificial intelligence, image processing, and pattern recognition. According to predictions, CAD systems will increase diagnostic rates, decrease operator dependence, and reduce the cost of medical auxiliary modalities^9–11^. Since false positive rates can result in inefficient therapy as well as psychological, physical, and monetary costs, it may help to reduce them. Additionally, it can stop false negative results that might result in skipped treatments or remissions. Detection sensitivity without CAD is reportedly around 80%, and sensitivity with it is reportedly over 90%^12^. The results showed that mammography combined with CAD provided 100% sensitivity for malignancies presenting as microcalcifications and 86% sensitivity for other mammographic cancer presentations. Consequently, CAD has become the most productive field of study in medical imaging for enhancing diagnosis accuracy^13,14^.

The foundational effect can be witnessed in the determination of breast cancer by examining breast Magnetic Resonance Imaging (MRI) data. It has an edge over other imaging diagnosis techniques due to its high responsiveness and no harmful emission^4^. MRI is subjected to imaging irregularities in the form of motion artifacts, noise, etc., which obstructs the segmentation process and ROI detection results are less accurate. In order to get accurate outputs, image registration positively impacts by providing non-linear ordered images and a crucial step in Dynamic Contrast-Enhanced MRI (DCE-MRI) based breast tumor diagnosis^15^. Apart from classical image segmentation algorithms, a number of machine learning (ML) techniques have contributed to automating the segmentation process with minimum human input, some of which are mentioned in the Related Work section and are helpful for the determination of cancer lesions. The development of the Computer-Aided Diagnosis (CAD) system which is rooted in modern digital visualization, pattern identification, and artificial intelligence, has helped to reduce flawed output, which results in pointless reception and wrong therapy. The most commonly used machine learning techniques are: Support Vector Machine (SVM) and Artificial Neural Network (ANN)^16^. Additional efforts have been made in building hybrid systems in order to obtain better sensitivity and accuracy. These include the frequently used CAD systems and some open-source applications that are included in a framework where the different models interact with each other for the automatic determination of cancerous lesions.

Deep neural networks are now trendy machine learning designs around numerous disciplines and are extensively stationed in research and related enterprises. In the medical visualization field, deep learning is mostly applied in the form of convolutional neural networks (CNNs) and its use in clinical practice has been rapidly growing over the years in order to refine the methodology of cancer identification. Deep learning has a huge list of applications in medical science. Their successful application to various datasets not only improves accuracy and precision in the study results but also significantly reduces the intervention of humans in a subjective manner. Radiotherapy, PET-MRI attenuation correction, and image registration are some of the deep learning applications in the medical imaging discipline. In the case of MRIs, deep learning is usually centered on fractionalization and categorization of rejuvenated intensity images^17^.

U-shaped Deep CNN model, namely U-Net^18^ is commonly implemented in biomedical image segmentation. One main advantage of U-Net is that it can be trained with a few images. Piantadosi et al.^19^ proposed a Three Time Points U-Net (3TP U-Net) for lesion segmentation in breast DCE-MRI and this proposal was evaluated using exclusive metadata consisting of coronal T1-weighted FLASH 3D breast DCE-MRI data. After reviewing rigorous existing literature, we found that there is no deep investigation of different U-Net models in breast lesion detection using DCE-MRI. This research intends to advance the state of the art and conduct deeper analysis regarding the application of U-Net models in lesion detection in women’s breast DCE-MRI. For this, U-Net models are selected: UNet, Dense UNet Attention UNet, UNet++, MultiResUNet, RAUNet, Inception U-Net, U-Net GAN. The models are evaluated on 100 slices of 20 women’s breast 2D Sagittal T2-Weighted fat-suppressed DCE-MRI. For comparative study purposes, V-Net, W-Net, and DeepLabV3+ are also taken into account in the experiment to figure out if U-Net models are capable of overcoming them. The effectiveness of the reviewed approaches was evaluated using seven performance metrics: accuracy, sensitivity, specificity, precision, geometric mean, F-measure, and false-positive rate. To demonstrate the effectiveness of U-Net models, the acquired results are analyzed using the non-parametric statistical test using Wilcoxon Sign Rank Test (WSRT)^20^, and are also further examined using the MCDA approach namely Technique for Order of Preference by Similarity to Ideal Solution (TOPSIS)^21^ to find out the best model.

### Contributions of this article

The following is a summary of the contributions:

1. Comprehensive investigations evaluating several U-Net models in lesion detection in women breast DCE-MRI.
2. A set of important metrics, such as accuracy, sensitivity, specificity, precision, F$_1$-score, geometric-mean, DSC, and False-Positive Rate (FPR), were utilized to analyze the results of experiments with 100 slices of women’s breast DCE-MRI.
3. The performances of different U-Net models are compared with the V-Net, W-Net, and DeepLabV3+ in this study.
4. Discussion of both quantitative and qualitative outcomes using statistical and multi-criteria decision-making techniques.

## Related works

So far, several mechanisms (Classical methods, Machine Learning methods, Optimization techniques, Fuzzy systems, and Hybrid systems) have been proposed for Breast MRI segmentation and lesion classification. Related works are discussed here.

### Classical image segmentation techniques

Priya et al.^22^ designed a biomedical surveillance apparatus ‘LabVIEW’ to pinpoint the accurate location of cancerous lumps from MR and CT scan images. Recognition of cancerous cells is carried on with the help of the Watershed Algorithm. To explore the practicality of registering ways delicate to time-to-peak (T(peak)) non-uniformity in order to check cancerous cells on breast DCE-MRI, a Time-To-Peak Optimization analysis was done by Fang et al.^23^, where the authors concluded distribution of T(peak) optimization can applied in judgment of diagnostic achievement, and be used as standard delicate to intra-lesion T(peak) non-uniformity. Si and Mukhopadhyay^24^ developed a breast DCE-MRI segmentation method using modified hard-clustering with Fireworks Algorithm (FWA) for lesion detection. Denoising of the MR images is completed using an anisotropic diffusion filter, while intensity inhomogeneities (IIHs) are corrected using the max filter-based method. The authors carried out the segmentation step using a hard-clustering technique with the FWA algorithm. Also, Si and Kar^25^ proposed a sectionalization technique using an altered hard-clustering technique with a multi-verse optimizer (MVO) for determining breast lesions in DCE-MRI. Preprocessing steps are similar to that of earlier work, then clustering technique is used for segmentation purposes. Finally, in the post-processing step, lesions are extracted. Patra et al.^26^ proposed a multi-level thresholding using Student Psychology-Based Optimizer (SPBO) for lesion detection and segmentation of DCE-MR images. Denoising of the breast MRIs was carried out with an Anisotropic diffusion filter followed up with Intensity Inhomogeneities correction. The processed images underwent segmentation assisted by the SPBO algorithm. It recorded an accuracy level of 99.44%, sensitivity of 96.84%, and DSC of 93.41%.

### Statistical Image Segmentation Techniques

A Markov Random Field (MRF) model and dissection approach rooted in the Bayesian theory of maximizing the posterior probability given by Wu et al.^27^ had an edge over and proved to be powerful in image accession. Furthermore, a new MRF model developed by Azmi et al.^28^, resolved the issue of complexity of computation by erasing out the need to use a monotonous approach and yielded Area Under the Curve (AUC) = 0.9724 and Mean Accuracy = 93.10%.

### Deep learning based segmentation techniques

A comparative study of Conventional and Multi-State Cellular Neural Networks was performed by Ertas et al.^29^ for breast region segmentation. The results of segmentation achieved by Conventional CNNs : (PR = $85.5\pm 17.0\%$, TPVF = $90.4\pm 17.8\%$ and FPVF = $2.0\pm 2.2\%$) and Multi-State CNNs: (PR = $99.3\pm 1.8\%$, TPVF = $99.5\pm 1.3\%$ and FPVF = $0.1\pm 0.2\%$). A novel technique (3D-LESH) was developed by Summrina et al.^30^ to determine breast lesions in volumetric medical images. Their results show that 3D-LESH helps in detecting various cancer stages. A CAD system designed by Rasti et al.^31^ is derived from a mixture ensemble of CNN (ME-CNN) to distinguish cancerous and non-cancerous breast tumors. In that study, the dataset consisted of T1-weighted axial image metadata over the entire breast region obtained with the image size of $512 \times 512$ pixels. The ME-CNN design had three CNN experts and a convolutional gating network which recorded a high validity of 96.39%, a responsiveness of 97.73%, and a specificity of 94.87%. Another CNN-based method developed by Xu et al.^32^ was used to slice the mammary gland region in transverse fat-suppressed breast DCE-MRI. They used 50 3-D fat-suppressed DCE-MRI series of datasets. The DSC was found to be 97.44%, DDC recorded 5.11%, and the distance error (automated fragmentation comparison with manual one) was about 1.25 pixels. Zhang et al.^33^ proposed an upgraded nine-layer CNN on a dataset of uni-breast mammogram images ($1024 \times 1024$) which resulted in the correctness of disease = 93.4%, correctness without a disease = 94.6%, exactness of 94.5%, and accuracy of 94.0%. A suitably modified CNN to fully automatize the significant mammary gland cell dissection job in 3D MR data (3D coronal DCE T1-weighted images), was given by Piantadosi et al.^34^. Mask-guided hierarchical learning (MHL) structure was developed by Zhang et al.^35^ for breast lump dissection via fully convolutional networks (FCN). An architecture rooted in U-net fCNN was introduced by Benjelloun et al.^36^, who worked on the idea of an upgraded Fully CNN (fCNN) by including regular fCNN layers and ensuing up-sampling ones to surge the image size and integrating earlier feature maps for a better image representation learning. Deep Learning assisted Efficient Adaboost Algorithm (DLA-EABA) for breast tumor determination was put forward by Zheng et al.^37^ with modern computational methods recorded a high accuracy level of 97.2%, Sensitivity 98.3%, and Specificity 96.5%. Negi et al.^38^ put forward a Generative Adversarial Network (GAN) based model operating on Breast Ultrasound (BUS) Images which consists of two modules: Residual-Dilated-Attention-Gate-UNet (RDAU-NET), which serves as dissolution block, and a CNN, which acts as distinguisher. This hybrid model is also named as WGAN-RDA-UNET. The overall Accuracy, PR-AUC, ROC-AUC and F1-score were achieved 0.98, 0.95, 0.89, and 0.88, respectively. A novel attention-guided joint-phase-learning network for multilabel segmentation method was developed by Qiao et al.^39^, which produced a Dice Coefficient of 0.83. Zhang et al.^40^ developed a deep learning approach using Mask Regional-CNN for automatic determination of breast lesions in DCE-MRI datasets (non-fat-sat sequence and fat-sat sequence). The tumor is segmented using a fuzzy c-means clustering algorithm and then Mask R-CNN is implemented. Their results show high accuracy in detecting and localizing the tumor.

An ailing administered deep learning technique for breast MRIs was evaluated in a study given by Liu et al.^41^. The evaluation was done in the absence of pixel-level segmentation in order to achieve higher specificity in breast lesion classification. The model achieved an AUC of 0.92 (SD $\pm 0.03$), accuracy of 94.2% (SD $\pm 3.4$), specificity of 95.3% (SD $\pm 3.3$), and sensitivity of 74.4% (SD $\pm 8.5$). Huo et al.^42^ presented a study to achieve higher accuracy and efficiency in the segmentation of whole breast in 3-D fat-suppressed DCE-MRI by introducing an adaptable deep learning framework using nnU-Net. Results show its robustness in achieving higher accuracy and prove to be a potential asset in clinical workflow in quantifying breast cancer risk. To surmount the weakness of regular imaging techniques used in machine learning-based approaches, such as limited data size and less information to feed them, Venkata and Lingamgunta^43^ introduced a CNN (CNN (LeNet-5)) based diagnosis of breast using Zenker moments which achieved 88.2% sensitivity and 76.92% accurateness, 83.3% sensitivity and 62.5% malignant growth accuracy. Jaglan et al.^44^ developed a one-ordered algorithm to distinguish breast lesions (normal/abnormal), which consists of an integrated fining technique for de-noising, breast boundary region extraction via selection of nipple and mid-sternum points, and followed by morphological operations and hole filling. For classification, an SVM was implemented in their study. The proposed method recorded an accuracy of 93.7%, sensitivity of 95.6%, and specificity of 87.2%.

Kim et al.^45^ developed an edge extraction algorithm (eLFA algorithm) over ultrasonic breast images which implements a cRNN-based learning design to distinguish breast lumps among others. The proposed model advances in most scenarios as an unnecessary specific set of limitations is avoided in detecting line segments and calculates threshold values itself to determine precise line segments masterfully, thereby achieving the highest accuracy of 99.75%. Lv et al.^46^ developed a dynamic mode-based self-supervised dual attention deep clustering network (DADCN) in order to attain detailed segmentation of breast intra-tumor heterogeneity region individually. The graph attention network learns and combines particular representations with features taken out from deep CNN. Soleimani et al.^47^ developed an algorithm that makes capital of Dijkstra’s process, and allows it to track the boundary between the pectoralis muscle and breast tissue as well as between the breast and its surroundings. By evaluating the proposed method, the authors reported its robustness and accuracy in nature.

## Methods

### DCE-MRI dataset

Total 100 T2-Weighted Sagittal fat-suppressed DCE-MRI 2D slices of 20 women patients were collected from public dataset of The Cancer Genome Atlas Breast Invasive Carcinoma (TCGA-BRCA)^48,49^ in “The Cancer Imaging Archive (TCIA), USA” (https://www.cancerimagingarchive.net). The size of all MRI slices is $256\times 256$. The ground truths are prepared using manual segmentation by the expert radiologist, which is considered as the gold standard^50^. The lesion pixels are assigned the true value and all other pixels are assigned false in the ground truth images.

### Overview of the work

In this work, lesion segmentation in breast DCE-MRI using V-Net, W-Net, and ten U-Net based deep learning models is proposed. The proposed method has the following steps.

Step 1: training all the proposed models for lesion segmentation.

Step 2: testing of the trained models for the test data and the generation of segmentation outputs.

Step 3: Localize the lesion in MRI.

**Figure 1:**
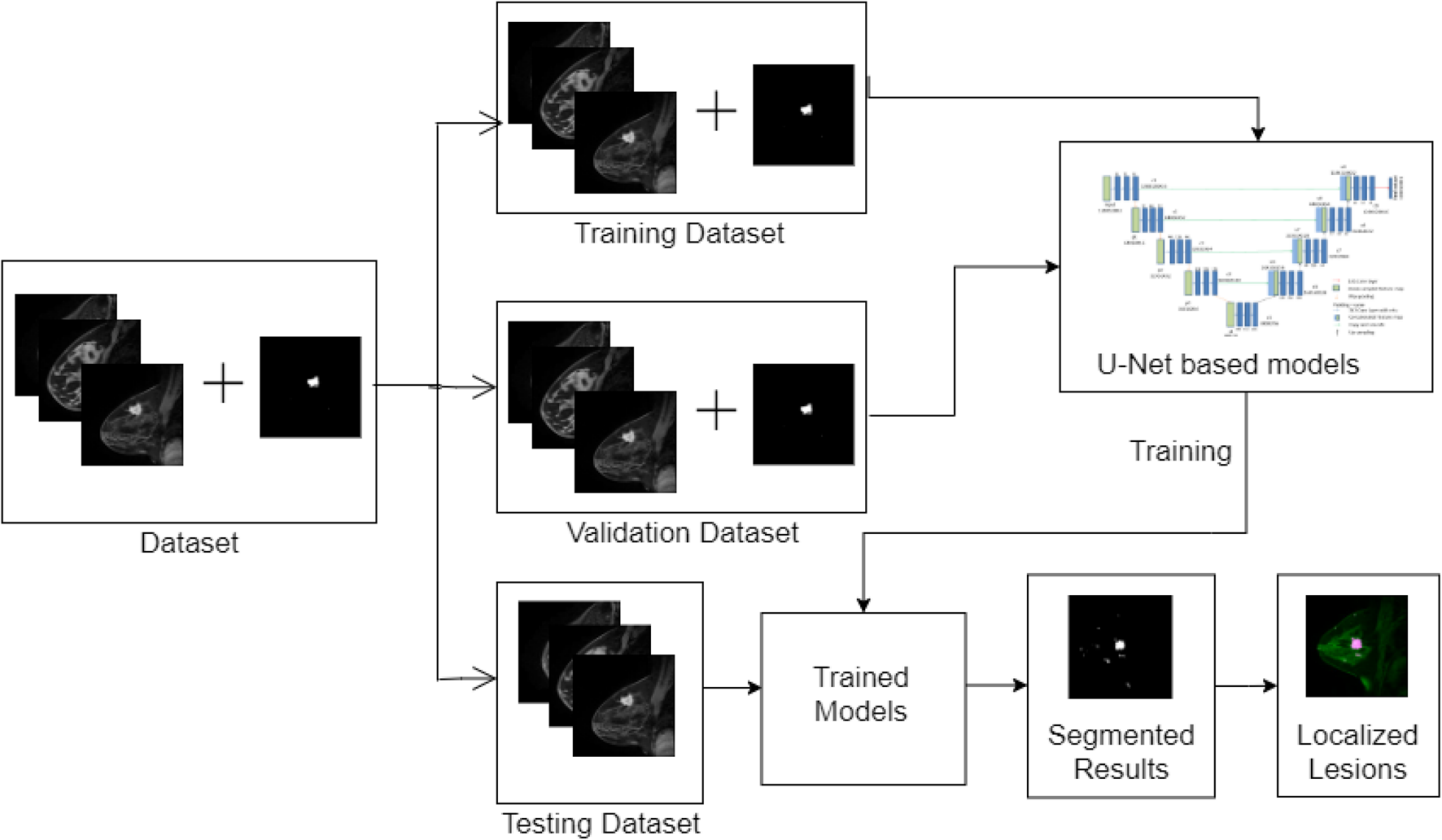
Overview of our proposed method.

The overview of the proposed method is provided in Fig. 1.

### Deep learning for segmentation

Deep learning models are being widely used in image segmentation as they are robust for performing such tasks. In image segmentation, a digital image is broken down into various segments and the targeted region is extracted. Segmentation tasks can be subdivided into two categories: semantic segmentation and instance segmentation. Semantic segmentation involves assigning a class label for every pixel in the given image. In our study, each pixel is divided into two classes. For each pixel, deep learning models need to determine if it forms a lesion on the breast tissue. In our study, V-Net, W-Net, U-Net and its nine variants are included to achieve the objectives.

#### U-net for segmentation

In image segmentation based on deep learning approaches U-Net is considered to be an efficient model that works well even with a limited number of training samples. The ’U’ shaped architecture of U-Net is symmetrical and has two major sections, which are the contracting network and expanding network. Down-sampling and up-sampling of the image are performed by these networks respectively. The contracting network is constituted by the general convolutional process whereas the expanding network is constituted by transposed 2D convolutional layers. Both down-sampling and up-sampling occur in a layer-wise manner. This architecture does not contain any fully connected layers.

**Figure 2:**
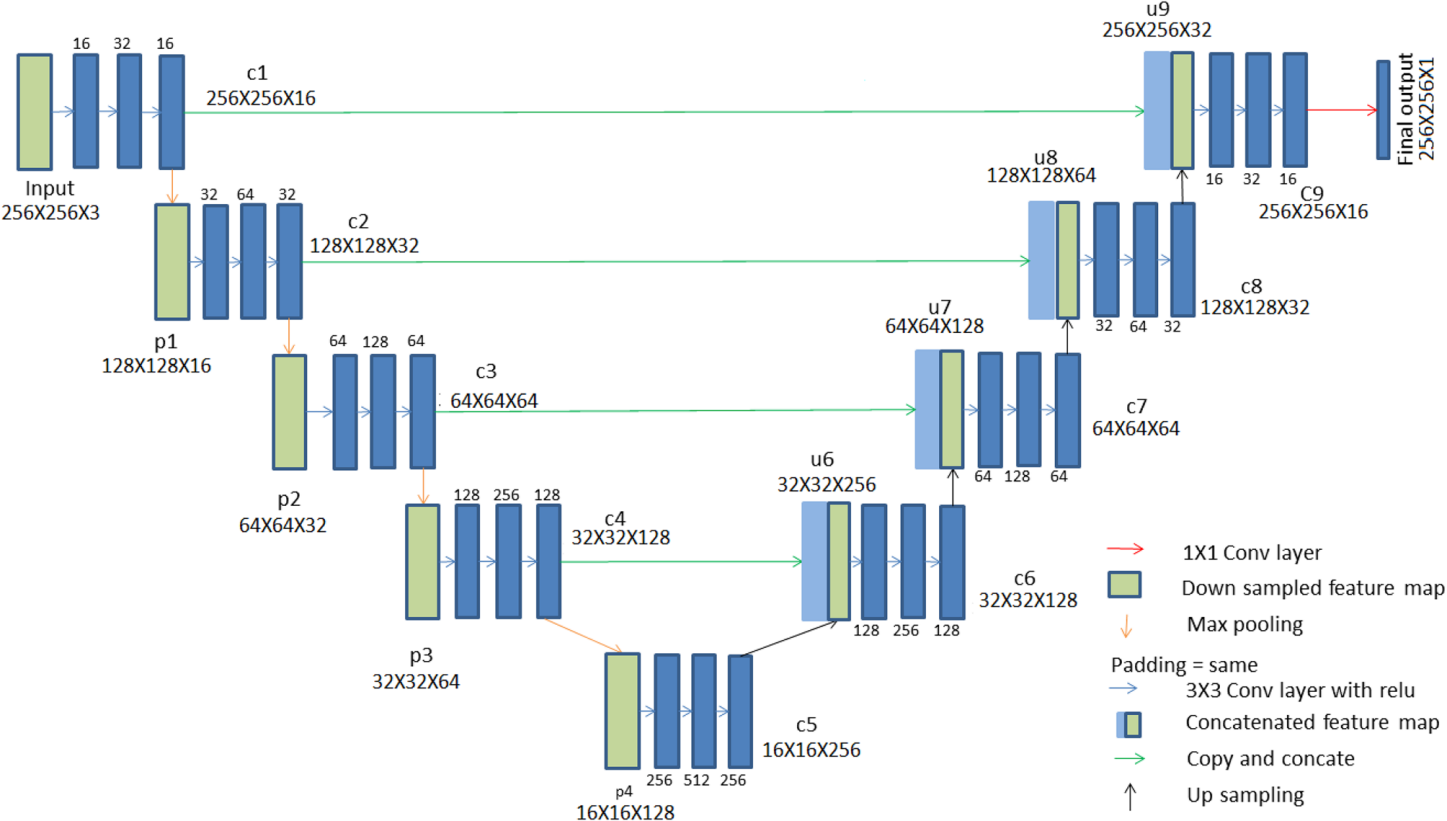
Architecture of U-Net. Each box in the diagram represents a multi-channel feature map.

The architecture of the classical U-Net model is presented in Fig. 2 and the interested readers are directed to respective articles of the different U-Net models for the network architecture diagrams. . Each of the contracting path and expanding path contains four sections. To link these two paths, there is another section of convolutional layers in the middle. In the contracting path, each section contains three padded $3\times 3$ convolution layers, that maintain the input dimensions. Out of these layers, the middle layer contains twice the number of feature channels than the other two layers. In each section, batch normalization layer followed by a rectified linear unit is added after each of the three convolution layers. For down-sampling in the end of each section, a $2\times 2$ max pooling operation is performed with stride 2. In such down-sampling activity, the feature channels get doubled. The transitional section that connects the contracting path and the expanding path contains similar convolution layers. Rectified linear units are added after this section. The sections in both the contracting path and expanding path follow the same feature channel increment pattern. The main purpose of the expanding path is upsampling. At each section of the expanding path, the feature map gets upsampled. This is followed by a $2\times 2$ convolution layer for reducing the number of feature channels by half. Then, a concatenation operation is performed between feature channels and the corresponding feature map from the contracting path. For maintaining the number of feature channels that have exactly the same format as that of the sections in the contracting path, the concatenation operation is followed by three $3\times 3$ padded convolution layers. Each $3\times 3$ convolution layer in each section of expanding path is followed by batch normalization and a rectified linear unit. A $1\times 1$ convolutional layer is added at the end to decide the class for each feature vector. The complete architecture has a total of 32 convolutional layers. U-Net’s architecture, which includes a contracting path for capturing context and a symmetric expanding path for accurate localization, is what gives it its competitive edge. With this layout, accurate segmentation outcomes are possible even with small training data.

We have implemented several other models: ResUNet, Dense UNet Attention UNet, UNet++, MultiResUNet, RAUNet, Inception U-Net, U-Net GAN. The architectures of these models are based on the derivation of the U-Net for multiple purposes.

ResUNet refers to Deep Residual U-Net. It is a semantic segmentation model based on U-Net and inspired by the idea of deep residual learning. This model was proposed for road area extraction. There are two major advantages of this model. First, the training process of deeper models requires more resources; therefore, residual units used in ResUNet ease the training process. Second, the networks can be designed with fewer parameters, which helps maintain improved performance due to the rich skip connections. This also facilitates information propagation.

A densely connected convolutional network strengthens the utilization of features and provides improved segmentation results even with a limited number of training samples. We combine the U-Net network and densely connected convolutional network (commonly called Dense UNet). Between each pair of convolutional layers there are some extra concatenation layers compared to U-Net and other traditional convolutional networks. In Dense UNet, each layer can acquire the feature maps of all its previous layers as inputs while its feature maps can be passed to all successive layers, and without increasing the size of datasets a higher segmentation accuracy can be achieved.

DUNet (Deformable U-Net) was initially proposed for retinal vessel segmentation. In DUNet, some of the convolutional layers of the basic U-Net are replaced with deformable convolution blocks. Upsampling operators are used to increase the output resolution. DUNet performs quite well in precise localization by combining low-level feature maps with high-level ones.

In Attention U-Net, attention gates are attached on top of the standard U-Net. It aims at increasing segmentation accuracy even with lesser training data. Attention gates reduce the computational resources wasted on irrelevant activations by highlighting only the relevant activations during training. This model was first applied to medical image segmentation which eliminates the necessity of applying an external object localisation model.

UNet++ was proposed for image segmentation in the field of medical science. It is encoder-decoder based network containing encoder and decoder sub-networks. The two sub-networks are connected by a series of nested, dense skip pathways. The re-designed skipping pathways also help to reduce the semantic gap between the feature maps of the encoder and decoder sub-networks.

In MultiResUNet, each pair of convolutional layer pairs in the basic U-Net is replaced with a MultiRes block. This configuration basically is derived from factorizing $5\times 5$ and $7\times 7$ convolution operations to $3\times 3$ ones and reuses them to obtain results from $3\times 3$, $5\times 5$ and $7\times 7$ convolution operations simultaneously. To regulate the semantic distance between Encoder and Decoder networks residual path is added. To proportionate the anticipated gap between two corresponding layers additional convolutions are added along the shortcut path.

RAUNet was initially proposed for the semantic segmentation of Cataract Surgical Instruments. The basic architecture of RAUNet is similar to that of U-Net. By combining both low and high-level feature maps, RAUNet extracts contextual information. To adaptively change the attention-aware features attention residual modules are integrated.

Inception U-Net is inspired by Inception net. Inception modules are introduced into the original U-Net architecture. This architecture is also encoder-decoder based. Inception modules are used because of the fact that convolution layers of different dimensions can correlate with different spatial features present in the same feature map. Inception U-Net follows U-net like architecture. However, here inception modules are used in place of the regular stack of convolution layers as in U-Net.

U-Net GAN uses a segmentation network as the discriminator. This segmentation network predicts two classes. In doing so, the discriminator gives the generator region-specific feedback.

Applications of all the models are mentioned in Table 1.

**Table 1:** Different U-Net models and their applications.

Model	Application	Refs.
U-Net	Segmentation of neuronal structures in electron microscopic stacks	^18^
ResUNet	Semantic segmentation of remotely sensed data	^51^
Dense UNet	Semantic segmentation with a small number of samples	^52^
DUNet	Retinal vessel segmentation	^53^
Attention U-Net	Multi-class CT abdominal segmentation	^54^
UNet++	Segmentation of cell nuclei, colon polyp, liver and lung nodule	^55^
MultiResUNet	Multimodal biomedical image segmentation. Used datasets are Fluorescence Microscopy, Electron Microscopy, Dermoscopy, Endoscopy and MRI	^56^
RAUNet	Semantic Segmentation of Cataract Surgical Instruments	^57^
Inception U-Net	Brain magnetic resonance imaging (MRI) tumor segmentation	^58^
U-Net GAN	Used to achieve detailed per-pixel feedback while sustaining the global coherence of synthesized images, by providing the global image feedback as well	^59^
V-Net	V-Net was first used to detect prostate segments from MRI volumes.	^60^
W-Net	W-Net is a W-shaped unsupervised image segmentation model.	^61^

#### V-net for segmentation

V-Net is a CNN-based deep learning model widely used in the field of medical image segmentation. This model was first used to detect prostate segments from MRI volumes.

The V-Net network consists of two paths, a compression path and an expansion path. The compression path compresses the signal, while the expansion path decompresses it to its original size. At each stage of the compression path, a residual function is learnt and a convolution operation is performed. At each stage of the expansion path, a deconvolution operation is performed. Similar to the compression path, residual function is learnt at each stage. Horizontal connections help to forward the features learnt in the early stages of the compression path to the expansion path.

#### W-net for segmentation

W-Net is an unsupervised image segmentation model consisting of an encoder network and a decoder network. Both the encoder and decoder networks are concatenated together to form an auto-encoder.

W-Net is a W-shaped architecture consisting of 18 modules. Each module contains two $3\times 3$ convolutional layers, one Rectified Linear Unit (ReLU), and batch normalization. The whole network contains 46 convolutional layers. The initial nine modules combined form the encoder unit that facilities the image segmentation, while the decoder unit consists of the later nine modules that form the reconstructed images.

## Results

In this paper, lesion detection using V-Net, W-Net, and ten U-Net based deep learning models is proposed. A summary of the parameters used in the models and hyperparameters for each model and the experimental environment of Google Colaboratory^62^ is presented in Tables 2 and 3 , respectively.

**Table 2:** Summary of all the parameters and hyperparameters used for each model.

Model	Loss function	Optimizer	Learning rate	Epochs	Batch size		
					Train	Test	Validate
U-Net	Binary cross-entropy	Adam	0.01	500	60	20	20
ResUNet	Binary cross-entropy	Adam	0.01	350	60	20	20
Dense UNet	Binary cross-entropy	Adam	0.01	250	60	20	20
DUNet	Binary cross-entropy	Adam	0.01	200	60	20	20
Attention U-Net	Binary cross-entropy	Adam	0.01	150	60	20	20
UNet++	Binary cross-entropy	Adam	0.01	245	60	20	20
MultiResUNet	Binary cross-entropy	Adam	0.01	300	60	20	20
RAUNet	Binary cross-entropy	Adam	0.01	200	60	20	20
Inception U-Net	Binary cross-entropy	Adam	0.01	85	60	20	20
U-Net GAN	Binary cross-entropy	Adam	0.01	1150	60	20	20
V-Net	Binary cross-entropy	Adam	0.01	600	60	20	20
W-Net	Binary cross-entropy	Adam	0.01	120	60	20	20
DeepLabV3+	Binary cross-entropy	Adam	0.01	10	60	20	20

**Table 3:** Experimental environment.

Name	Configuration
GPU	Tesla T4
GPU Size	15109MiB
Python	Version 3.10.12
CUDA Version	11.2

We compare the effectiveness of V-Net, W-Net, and U-Net based deep learning models on the basis of evaluation metrics: Accuracy, Precision, Sensitivity, F-measure, Specificity, Geometric Mean (G-mean), DSC, and false positive rate (FPR)^63^. Let TP, FP, TN, and FN be the True Positive rate, False Positive rate, True Negative rate, and False Negative rate, respectively. Various performance measures used in our study can be calculated as follows:

1 $$\begin{aligned} Accuracy= & {} (TP + TN)/(TP + FN + TN + FP) \end{aligned}$$

2 $$\begin{aligned} Precision= & {} TP/(TP + FP) \end{aligned}$$

3 $$\begin{aligned} Sensitivity(recall)= & {} TP/(TP + FN) \end{aligned}$$

4 $$\begin{aligned} F - measure= & {} \frac{2 \times recall \times precision}{recall + precision} \end{aligned}$$

5 $$\begin{aligned} Specificity= & {} TN/(TN + FP) \end{aligned}$$

6 $$\begin{aligned} G - mean= & {} \sqrt{Sensitivity \times Specificity} \end{aligned}$$

For significant evaluation of the lesion segmentation performance, another widely used performance metric known as DSC is used. The formula for calculating DSC is:

7 $$\begin{aligned} DSC(A, B) = \frac{2|A \cap B|}{|A| \cup |B|} \end{aligned}$$

where A and B are representing binary masks for the segmented lesion and ground truth, respectively. DSC indicates the overlapping ratio between the segmented lesion and the ground truth. A higher DSC value indicates better lesion segmentation performance.

The problem in hand in this current work is to classify the pixels of breast MR images into lesions (cancerous tissues) and non-lesions (non-cancerous or healthy tissues). In the ground truth images, the pixels belonging to lesions are labeled as ‘1’ whereas pixels belonging to non-lesions are labeled as ‘0’. Similarly, in the resultant images during testing of the models, the lesional pixels are assigned to ‘1’ and non-lesional pixels are assigned to ‘0’. The loss function used for each model is binary cross-entropy loss. The equation for the binary cross-entropy loss can be written as follows:

$$\begin{aligned}L(Y_{i},\hat{Y_{i}})=-\frac{1}{N}\sum _{i=1}^{N}Y_{i}log(\hat{Y_{i}}) + (1-Y_{i})log(1-\hat{Y_{i}})\end{aligned}$$

Here, the number of training data points is defined by N, $Y_i$ is the ground truth label, and $\hat{Y_i}$ is the label predicted by the model.

The optimizer used is Adam. Adam optimizer updates the weight parameters such that loss function gets minimized. Keras’s default learning rate (0.01) is used in training. In addition, batch size of 60, 20 and 20 is used for training, validation, and testing, respectively. Each of the models is trained for different epochs based on hyperparameter tuning implemented in KerasTuner. Other hyperparameters are selected based on the Random Search^64^ method to achieve the best possible outcome from each trained model. Table 2 gives a summary of hyperparameters used for the models. All the models are trained on Tesla T4 GPU, with CUDA Version 11.2 and a GPU size of 15109 MiB. GPU configurations are mentioned in Table 3.

After training each model to the desired number of epochs, all 20 images from the test set are evaluated. For analysis, the mean and standard deviation of different performance measures over those 20 results are taken into consideration.

### Quantitative results

The performance measures for each model are briefly presented in Table 4. The box plots (models versus different performance measures) of different models over 20 MR images are shown in Figs. 1, 2, 3, 4, 5, 6, 7 and 8 in order to provide a better understanding of the results.

**Table 4:** Mean and standard deviation (in parenthesis) of performance measure values (in %) for all the models for the test set.

Performance matrix	U-Net	ResUNet	Dense UNet	DUNet	Attention U-Net	UNet++	MultiResUNet	RAUNet	Inception U-Net	U-Net GAN	V-Net	W-Net	DeepLabV3+
Accuracy	99.71	99.62	99.74	99.35	99.72	99.53	99.3	**99.76**	99.68	99.45	99.58	99.37	97.67
	(0.00274)	(0.00326)	(0.00252)	(0.00520)	(0.00265)	(0.00458)	(0.0138)	(0.00228)	(0.00241)	(0.00513)	(0.00205)	(0.00625)	(0.01016)
Precision	97.34	**99.56**	93.21	75.53	94.15	85.60	87.45	90.82	90.21	73.04	73.46	79.04	1.09
	(0.06230)	(0.00857)	(0.05579)	(0.10810)	(0.05087)	(0.10467)	(0.19029)	(0.07891)	(0.09152)	(0.25506)	(0.12827)	(0.12813)	(0.01605)
Sensitivity	71.99	62.26	78.93	63.22	75.92	73.88	72.27	**85.04**	74.90	52.46	83.23	68.91	0.73
	(0.24782)	(0.24961)	(0.26525)	(0.21946)	(0.28189)	(0.27925)	(0.29846)	(0.23257)	(0.23654)	(0.36507)	(0.20649)	(0.25570)	(0.00643)
F-measure	79.21	72.86	81.51	64.80	79.91	73.90	74.29	**85.04**	78.41	57.68	76.22	68.46	0.73
	(0.23444)	(0.24715)	(0.22790)	(0.16971)	(0.23059)	(0.20862)	(0.27395)	(0.16228)	(0.20153)	(0.33604)	(0.15155)	(0.18582)	(0.00748)
Specificity	99.98	**99.99**	99.91	99.65	99.76	99.84	99.73	99.91	99.90	99.87	99.73	99.60	98.78
	(0.00034)	(0.00005)	(0.00108)	(0.00494)	(0.00405)	(0.00146)	(0.00859)	(0.00075)	(0.00096)	(0.00173)	(0.00132)	(0.00650)	(0.00554)
G-mean	82.62	76.13	86.55	77.28	84.63	83.45	82.14	**90.97**	84.72	66.94	90.09	80.56	7.09
	(0.19239)	(0.20717)	(0.19838)	(0.17907)	(0.16532)	(0.20247)	(0.21552)	(0.14854)	(0.17438)	(0.27553)	(0.13505)	(0.19129)	(0.04706)
DSC	79.21	72.86	81.51	64.80	79.91	73.90	74.29	**85.04**	78.41	57.68	76.22	68.46	0.73
	(0.23444)	(0.24715)	(0.22790)	(0.16971)	(0.23059)	(0.20862)	(0.27395)	(0.16228)	(0.20153)	(0.33604)	(0.15155)	(0.18582)	(0.00748)
FPR	0.0181	**0.0032**	0.0857	0.3466	0.0509	0.1552	0.0468	0.0858	0.0974	0.1257	0.2658	0.3913	1.22
	(0.000342)	(0.000055)	(0.001085)	(0.004942)	(0.000499)	(0.001465)	(0.000461)	(0.000756)	(0.000969)	(0.001736)	(0.00132)	(0.00650)	(0.00554)

Bold-faced results indicate better.

**Figure 3:**
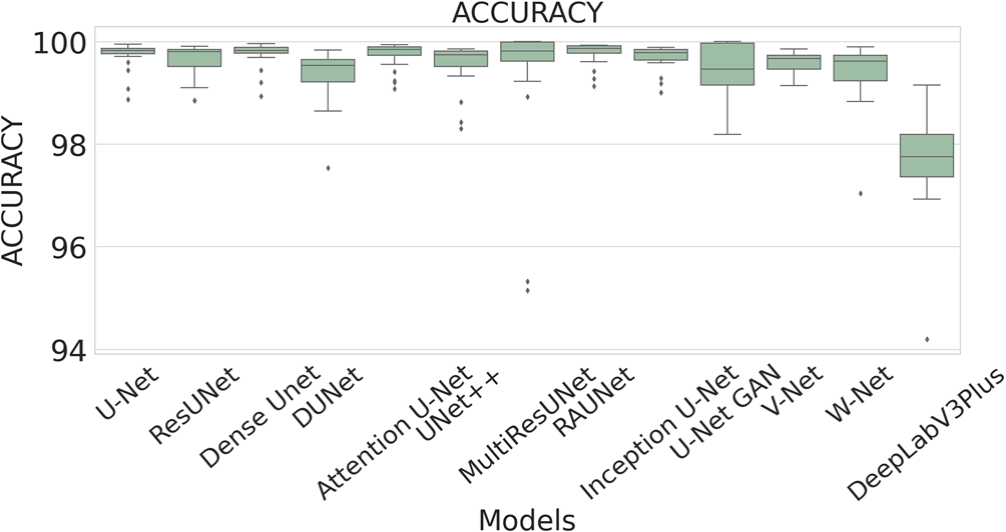
The box plot (models versus accuracy) of comprehensive classification performance of different models over 20 MR Images.

**Figure 4:**
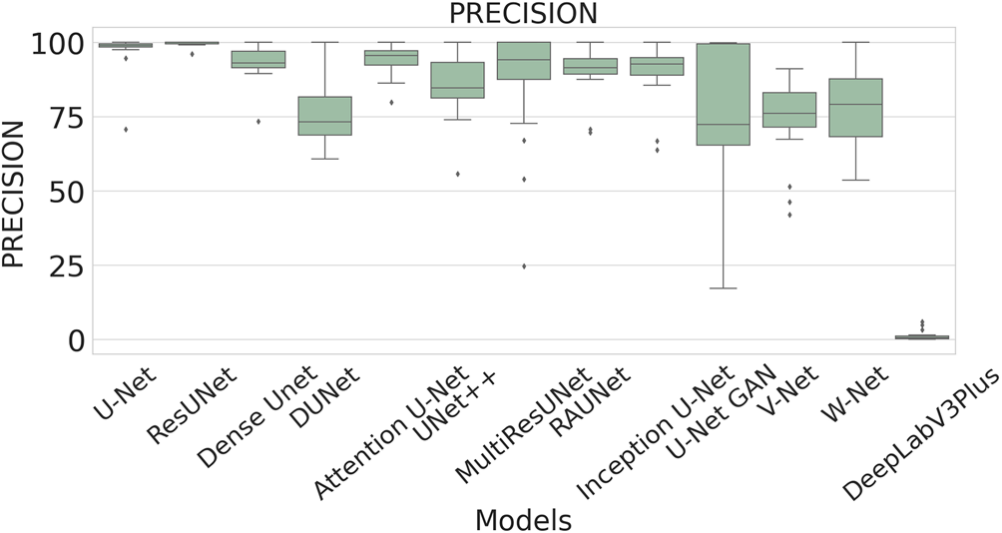
The box plot (models versus precision) of comprehensive classification performance of different models over 20 MR Images.

**Figure 5:**
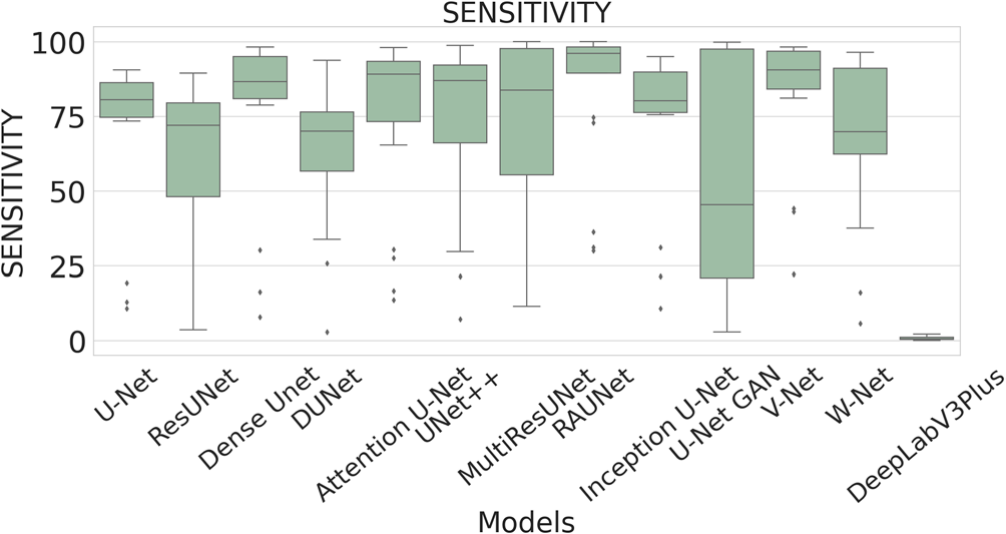
The box plot (models versus sensitivity) of comprehensive classification performance of different models over 20 MR Images.

**Figure 6:**
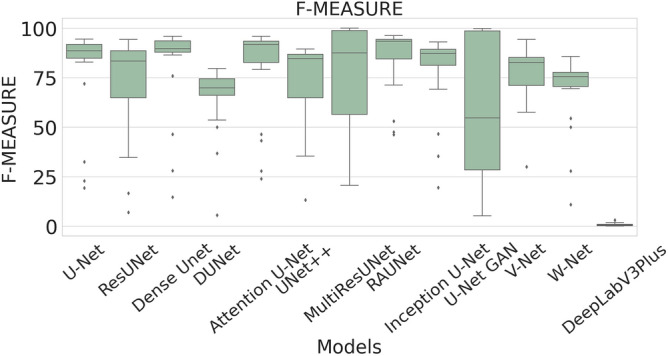
The box plot (models versus f-measure) of comprehensive classification performance of different models over 20 MR Images.

**Figure 7:**
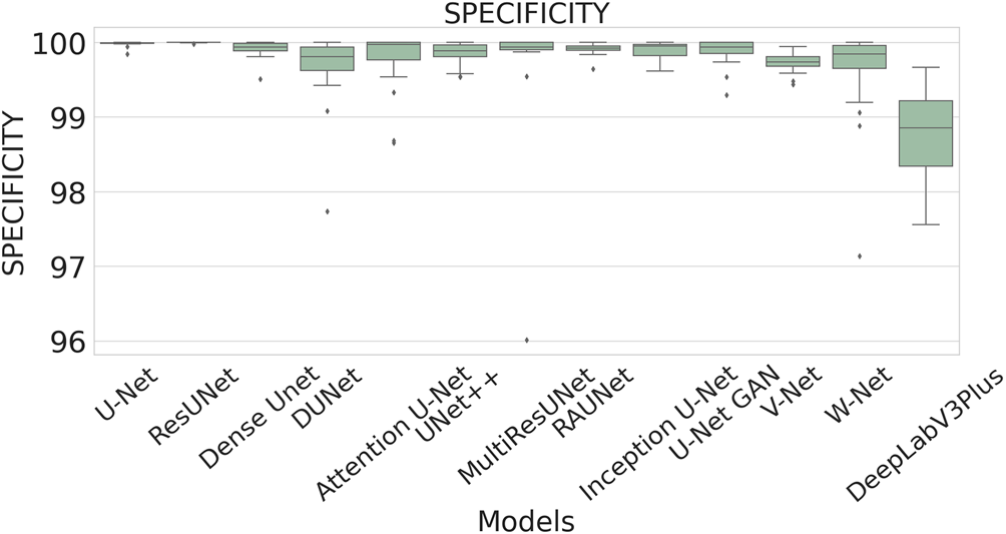
The box plot (models versus specificity) of comprehensive classification performance of different models over 20 MR Images.

**Figure 8:**
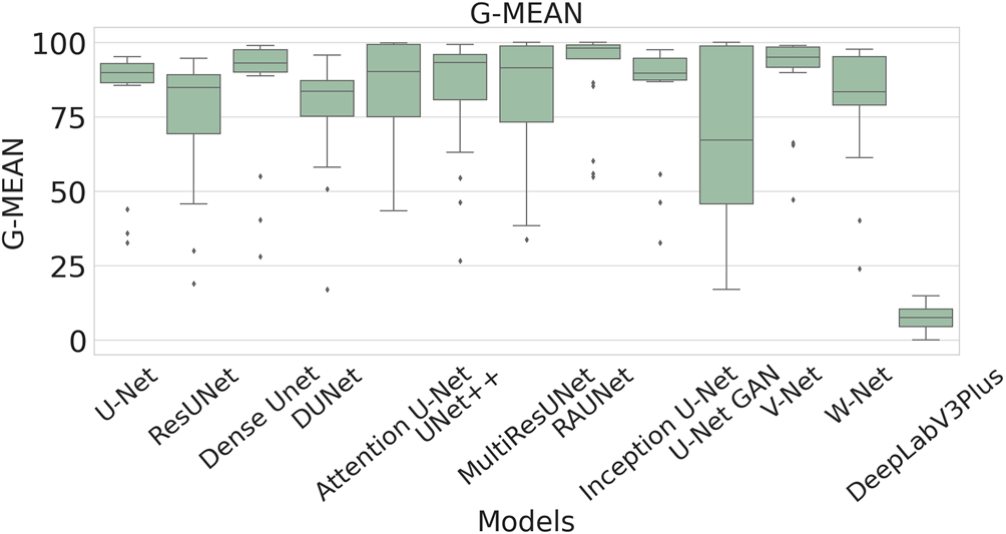
The box plot (models versus g-mean) of comprehensive classification performance of different models over 20 MR Images.

In Table 4, it is observed that RAUNet has achieved a higher mean classification accuracy than rest of the models. The mean accuracy of RAUNet is 99.76% with standard deviation of 0.00228, whereas mean accuracies the other models namely, U-Net, ResUNet, Dense UNet, DUNet, Attention U-Net, UNet++, MultiResUNet, Inception U-Net, U-Net GAN, V-Net, and W-Net are 99.71%, 99.62%, 99.74%, 99.35%, 99.72%, 99.53%, 99.30%, 99.68%, 99.45, 99.58%, and 99.37%, respectively.

The average precision value of the ResUNet model is 99.56%, which is the highest among all the models. Sensitivity is considered as one of the key measures in image segmentation. The mean sensitivity value of RAUNet is 85.04%, whereas the mean sensitivity values of U-Net, ResUNet, Dense UNet, DUNet, Attention U-Net, UNet++, MultiResUNet, Inception U-Net, U-Net GAN, V-Net, and W-Net are 71.99%, 62.26%, 78.93%, 63.22%, 75.92%, 73.88%, 72.27%, 74.90%, 52.46%, 73.46%, and 79.04% respectively. These values are relatively low. Though mean specificity value obtained from all the models are very high, ResUNet shows the highest mean specificity value of 99.99% with a standard deviation of 0.00005.

The F-measure summarizes precision and sensitivity into a single measure. The mean F-measure for the RAUNet model is 85.04% which is higher than F-measure values of U-Net, ResUNet, Dense UNet, DUNet, Attention U-Net, UNet++, MultiResUNet, Inception U-Net, U-Net GAN, V-Net, and W-Net. Since it is difficult to get a detailed view of the performance of the segmentation task from either precision or sensitivity, the usage of F-measure becomes beneficial in this regard. In our study the higher value of F-measure in the case if the RAUNet model indicates that this model is doing a very well job in lesion segmentation.

The RAUNet model has an excellent mean G-mean score of 90.97%, which is also higher than that of U-Net, ResUNet, Dense UNet, DUNet, Attention U-Net, UNet++, MultiResUNet, Inception U-Net, U-Net GAN, V-Net, and W-Net. Since the proportion of healthy tissues is very high compared to lesions in breast MR images, it implies high-class imbalance problem may be present. A high mean G-mean score indicates that the model is capable of solving this problem.

The mean DSC value of RAUNet is 85.04% which is higher than other models. DSC value reflects the extent of overlap of the result with the ground truths of the segmented lesions. A higher DSC value means better performance of the model.

FPR is a measure of how many samples are wrongly identified as the positive out of all the samples. FPR does not show any responsive nature towards changes in data distribution and, therefore, it can be used even with imbalanced data. The mean FPR of the ResUNet model is 0.0032% which is much lower than that of U-Net, Dense UNet, DUNet, Attention U-Net, UNet++, MultiResUNet, RAUNet, Inception U-Net, U-Net GAN, V-Net, and W-Net. The lower value of FPR in the model of ResUNet indicates that wrongly identified negative samples are minimum in images.

The robustness of the models is one of the most essential aspects of the segmentation task. A robust model can be expected to perform well irrespective of fluctuations in the data. It is measured on the basis of the standard deviation of different performance measures. Stronger robustness is indicated by lower standard deviation. From the standard deviation results of all the models in Table 3, it is difficult to find the most robust model as no model has the lowest standard deviations for all the measurements. Therefore, some statistical analysis methods are adopted to find the most robust model.

**Figure 9:**
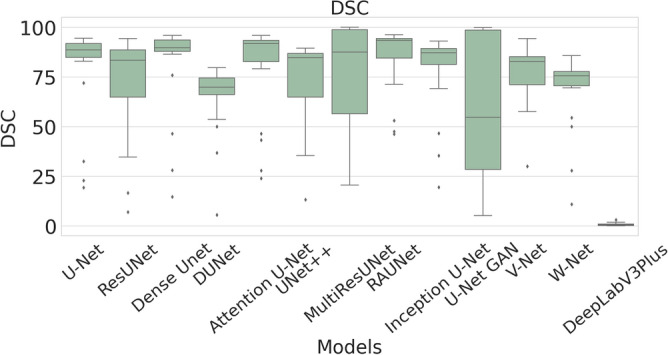
The box plot (models versus dsc) of comprehensive classification performance of different models over 20 MR Images.

**Figure 10:**
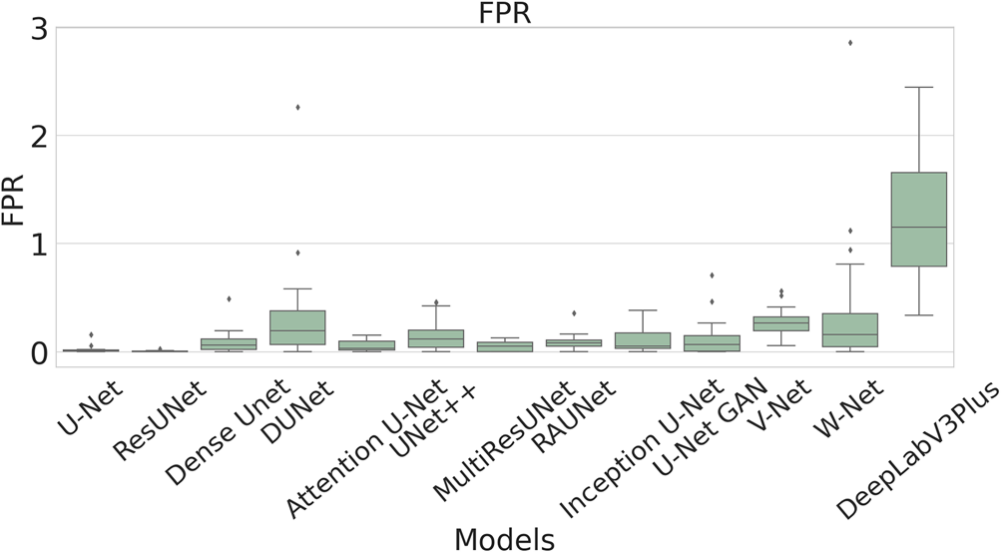
The box plot (models versus fpr) of comprehensive classification performance of different models over 20 MR Images.

A boxplot gives a good graphical interpretation of the diversity of values. Using boxplots, visual comparison of performance measures of different models can be easily examined. It is observed from Fig. 3 that the RAUNet has the highest median accuracy of classification compared to the other models. It also indicates that, in comparison to other models, the minimum and maximum accuracy values in the case of RAUNet is comparatively higher with the least difference between both the values. In Fig. 4, it can be observed that ResUNet has a higher median precision score than that of other models. Similarly, Fig. 5, shows that RAUNet has a higher median sensitivity score than the other models. Fig. 7 displays that ResUNet has a slightly higher median specificity score compared to other models. RAUNet has the highest median G-Mean, F-Measure, and DSC value, as shown in, Figs. 8, 6 and 9. From Fig. 10, it is observed that ResUNet has the minimum median FPR value. All together, as shown in Figs. 3, 4, 5, 6, 7, 8, 9 and 10, it is clear that no model has the highest median value for all the measurements.

#### Statistical analysis using Wilcoxon Signed Rank Test

The models’ effectiveness is assessed using a non-parametric test. The best models based on results in Table 4 are compared against the other models pair-wise using the Wilcoxon Signed Rank Test (WSRT) with significance level ($\alpha$) through IBM SPSS Statistics 23^65^ . The test results based on the accuracy, precision, sensitivity, F-measure, specificity, G-mean, DSC, and FPR are respectively described in Tables 5, 6, 7, 8, 9, 10, 11 and  12. From the WSRT results based on accuracy, sensitivity, F-measure, G-mean, and DSC, it is observed that the RAUNet model statistically outperforms other models except MultiResUNet for accuracy, VNet for sensitivity, VNet and Attention U-Net for G-Mean. On the other hand, the ResUNet model statistically outperforms all other models based on precision, specificity, and FPR.

**Table 5:** WSRT results on accuracy.

Pair	*p*-value	h	Pair	*p*-value	h
RAUNet vs. UNet	0.003182	1	RAUNet vs. MultiResUNet	0.061953	0
RAUNet vs. ResUNet	0.000103	1	RAUNet vs. Inception U-Net	0.001161	1
RAUNet vs. Dense UNet	0.040136	1	RAUNet vs. U-Net GAN	0.008962	1
RAUNet vs. DUNet	0.000681	1	RAUNet vs. V-Net	0.000140	1
RAUNet vs. Attention U-Net	0.013733	1	RAUNet vs. W-Net	0.000103	1
RAUNet vs. U-Net++	0.000163	1	RAUNet vs. DeepLabV3+	0.000089	1

**Table 6:** WSRT results on precision.

Pair	*p*-value	h	Pair	*p*-value	h
ResUNet vs. UNet	0.000351	1	ResUNet vs. RAUNet	0.000155	1
ResUNet vs. Dense UNet	0.000293	1	ResUNet vs. Inception U-Net	0.000196	1
ResUNet vs. DUNet	0.000132	1	ResUNet vs. U-Net GAN	0.000219	1
ResUNet vs. Attention U-Net	0.000293	1	ResUNet vs. V-Net	0.000089	1
ResUNet vs. U-Net++	0.000196	1	ResUNet vs. W-Net	0.000196	1
ResUNet vs. MultiResUNet	0.002611	1	ResUNet vs. DeepLabV3+	0.000088	1

**Table 7:** WSRT results on sensitivity.

Pair	*p*-value	h	Pair	*p*-value	h
RAUNet vs. UNet	0.000189	1	RAUNet vs. MultiResUNet	0.001162	1
RAUNet vs. ResUNet	0.000089	1	RAUNet vs. Inception U-Net	0.000593	1
RAUNet vs. Dense UNet	0.017583	1	RAUNet vs. U-Net GAN	0.000449	1
RAUNet vs. DUNet	0.001325	1	RAUNet vs. V-Net	0.184181	0
RAUNet vs. Attention U-Net	0.000593	1	RAUNet vs. W-Net	0.001116	1
RAUNet vs. U-Net++	0.000449	1	RAUNet vs. DeepLabV3+	0.000089	1

**Table 8:** WSRT results on F-measure.

Pair	*p*-value	h	Pair	*p*-value	h
RAUNet vs. UNet	0.001019	1	RAUNet vs. MultiResUNet	0.015240	1
RAUNet vs. ResUNet	0.000089	1	RAUNet vs. Inception U-Net	0.000390	1
RAUNet vs. Dense UNet	0.040044	1	RAUNet vs. U-Net GAN	0.004045	1
RAUNet vs. DUNet	0.001019	1	RAUNet vs. V-Net	0.001019	1
RAUNet vs. Attention U-Net	0.005111	1	RAUNet vs. W-Net	0.000103	1
RAUNet vs. U-Net++	0.000120	1	RAUNet vs. DeepLabV3+	0.000089	1

**Table 9:** WSRT results on specificity.

Pair	*p*-value	h	Pair	*p*-value	h
ResUNet vs. UNet	0.000349	1	ResUNet vs. RAUNet	0.000292	1
ResUNet vs. Dense UNet	0.000293	1	ResUNet vs. Inception U-Net	0.000195	1
ResUNet vs. DUNet	0.000132	1	ResUNet vs. U-Net GAN	0.000219	1
ResUNet vs. Attention U-Net	0.002703	1	ResUNet vs. V-Net	0.000089	1
ResUNet vs. U-Net++	0.000196	1	ResUNet vs. W-Net	0.000196	1
ResUNet vs. MultiResUNet	0.003438	1	ResUNet vs. DeepLabV3+	0.000089	1

**Table 10:** WSRT results on G-Mean.

Pair	*p*-value	h	Pair	*p*-value	h
RAUNet vs. UNet	0.000254	1	RAUNet vs. MultiResUNet	0.001162	1
RAUNet vs. ResUNet	0.000089	1	RAUNet vs. Inception U-Net	0.000517	1
RAUNet vs. Dense UNet	0.015240	1	RAUNet vs. U-Net GAN	0.000593	1
RAUNet vs. DUNet	0.001325	1	RAUNet vs. V-Net	0.156004	0
RAUNet vs. Attention U-Net	0.681322	0	RAUNet vs. W-Net	0.000892	1
RAUNet vs. U-Net++	0.000681	1	RAUNet vs. DeepLabV3+	0.000089	1

**Table 11:** WSRT results on DSC.

Pair	*p*-value	h	Pair	*p*-value	h
RAUNet vs. UNet	0.001019	1	RAUNet vs. MultiResUNet	0.015240	1
RAUNet vs. ResUNet	0.000089	1	RAUNet vs. Inception U-Net	0.000390	1
RAUNet vs. Dense UNet	0.040044	1	RAUNet vs. U-Net GAN	0.004045	1
RAUNet vs. DUNet	0.001019	1	RAUNet vs. V-Net	0.001019	1
RAUNet vs. Attention U-Net	0.005111	1	RAUNet vs. W-Net	0.000103	1
RAUNet vs. U-Net++	0.000120	1	RAUNet vs. DeepLabV3+	0.000089	1

**Table 12:** WSRT results on FPR.

Pair	*p*-value	h	Pair	*p*-value	h
ResUNet vs. UNet	0.000427	1	ResUNet vs. RAUNet	0.000293	1
ResUNet vs. Dense UNet	0.000293	1	ResUNet vs. Inception U-Net	0.000196	1
ResUNet vs. DUNet	0.000132	1	ResUNet vs. U-Net GAN	0.000218	1
ResUNet vs. Attention U-Net	0.000293	1	ResUNet vs. V-Net	0.000089	1
ResUNet vs. U-Net++	0.000196	1	ResUNet vs. W-Net	0.000196	1
ResUNet vs. MultiResUNet	0.003438	1	ResUNet vs. DeepLabV3+	0.000089	1

#### Multi-criteria decision analysis (MCDA)

Decision-making with multi-objectives is a difficult task and prone to errors. Multi-criteria decision analysis^21,66^ solves the issues of multi objectives up to a large extent and it has been widely used in operations research and management science. MCDA aims to provide the best reasonable solution based on the conditions provided. In practice, problems may be conflicting, and any solution may not meet all the criteria simultaneously. From the performance measures in Table 4 and their statistical analysis in Tables 5, 6, 7, 8, 9, 10, 11 and  12, it is observed that no single model could achieve the best results for all the performance metrics. Therefore, it is difficult to select the best one. In order to find out the best model, we have conducted a multi-criteria decision analysis. In our study, a prominent MCDA method namely Technique for Order of Preference by Similarity to Ideal Solution (TOPSIS) with Information Entropy Weighting Methodology^67^ is used for performance analysis. At present, MCDA is widely used in performance assessment of methods^26,68,69^. For our study, accuracy, precision, sensitivity, F-measure, specificity, G-mean, DSC, and FPR are used as multiple criteria. There is a conflict between FPR and other criteria. Lower values of FPR indicate better results, which is completely opposite to other criteria. MCDA ranks based on the TOPSIS technique are shown in Table 13. From Table 13, it is observed that the ResUNet model has the top rank followed by U-Net. W-Net has the lowest rank.

**Table 13:** Multi-criteria decision analysis ranks based on TOPSIS technique Information Entropy Weighting Methodology.

Methods	Score	Rank
ResUNet	0.9126	1
U-Net	0.3261	2
MultiResUNet	0.2896	3
Attention U-Net	0.2291	4
Dense UNet	0.1468	5
RAUNet	0.1178	6
Inception U-Net	0.1122	7
U-Net GAN	0.1108	8
UNet++	0.1083	9
V-Net	0.0919	10
DUNet	0.0860	11
W-Net	0.0825	12
DeepLabV3+	0.0759	13

### Visual results

**Figure 11:**
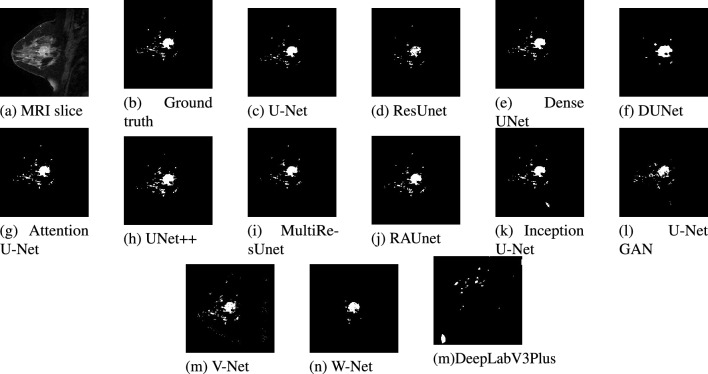
Results of different models for the data of Patient #1. (**a**) Original MR image. (**b**) Ground truth of MR image. (**c**–**n**) Segmented images using different models.

**Figure 12:**
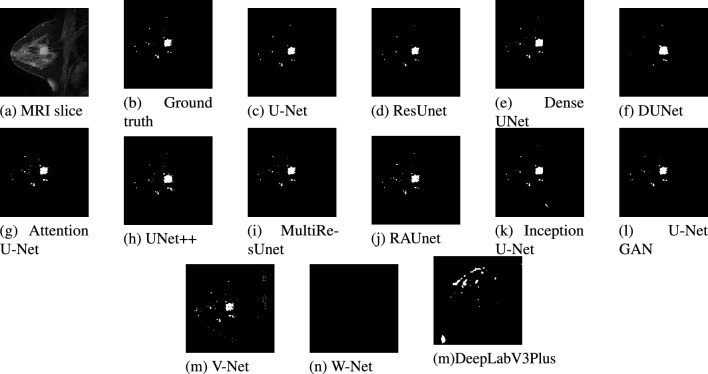
Results of different models for the data of Patient #2. (**a**) Original MR image. (**b**) Ground truth of MR image. (**c**–**n**) Segmented images using different models.

**Figure 13:**
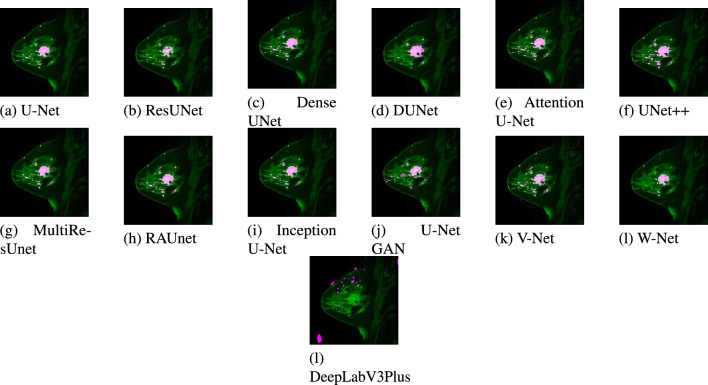
Localized lesions (bright colored spot) in MR images for Patient #1. (**a**) U-Net. (**b**) ResUnet. (**c**) DenseUnet. (**d**) DUNet. (**e**) Attention U-Net. (**f**) UNet++. (**g**) MultiResUnet. (**h**) RAUNet. (**i**) Inception U-Net. (**j**) U-Net GAN. (**k**) V-Net. (**l**) W-Net (**k**) DeepLabV3Plus.

**Figure 14:**
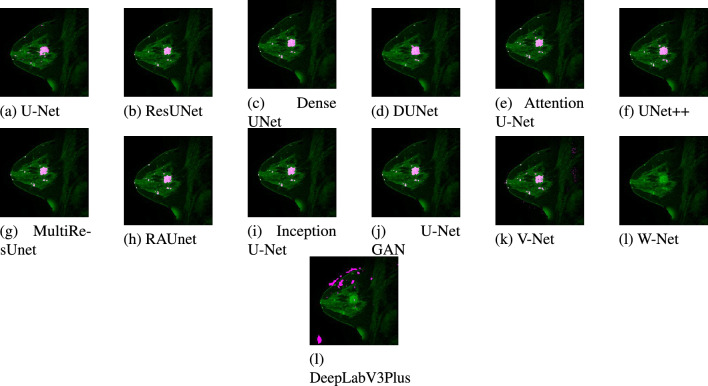
Localized lesions (bright colored spot) in MR images for Patient #2. (**a**) U-Net. (**b**) ResUnet. (**c**) DenseUnet. (**d**) DUNet. (**e**) Attention U-Net. (**f**) UNet++. (**g**) MultiResUnet. (**h**) RAUNet. (**i**) Inception U-Net. (**j**) U-Net GAN. (**k**) V-Net. (**l**) W-Net. (**k**) DeepLabV3Plus.

A test dataset consisting of 20 images has been used for the validation of the models. Due to space constraints, out of the 20 test results, only two images belonging to two patients are presented. The segmented results from each model for patient #1 and patient #2 are displayed in Figs. 11 and 12 respectively. Figures 13 and 14 show the localized lesions for patients 1 and 2 respectively in a comparative view.

For patient #1, Fig. 11, shows U-Net segmented result in Fig. 11c and ground truth in Fig. 11b are almost similar. The ground truth image and the U-Net segmented image contain 1071 and 935 numbers of lesion pixels, respectively, while the count of overlapping lesion pixels of both images is 931. This implies that U-Net is performing well in fully segmenting the lesions. Some parts of the lesions are missing from the image segmented by the ResUNet model as shown in Fig. 11d. Some dark spots can be visible inside the large lesion in the ResUNet segmented image. As shown in Fig. 11e, lesions are well segmented from the Dense UNet segmented image. This model performs well in the detection of the lesions. On the contrary, DUNet did not perform well in segmenting the lesions, as displayed in Fig. 11f. In addition, Fig. 11g–k, demonstrates that Attention U-Net, UNet++, MultiResUNet, RAUNet and Inception U-Net segmented images are mostly similar to the ground truth. Hence, these five models have good performance in the segmentation of lesions. Finally, from U-Net GAN, and V-Net segmented images in Fig. 11l,m in comparison with the ground truth image, we find their performance satisfactory, while W-Net segmented image in Fig. 11n, the performance is not satisfactory.

From Fig. 12, much similarity can be observed between the U-Net segmented result in Fig. 12c and the ground truth in Fig. 12b for patient #2. The ground truth image and the U-Net segmented image contain 516 and 474 numbers of lesion pixels, respectively, while the count of overlapping lesion pixels of both images is 467. The U-Net model is performing well in segmenting the lesions in the image. When we examined ResUNet and Dense UNet segmented images in Fig. 12d,e, we found that ResUNet and Dense UNet also perform well in segmenting the lesions. In DUNet segmented image in Fig. 12f, though lesions are quite well segmented, some healthy tissues are detected as lesions. DUNet fails to properly segment the lesions in the image. Fig. 12g, supports that, Attention U-Net has good performance in fully segmenting lesions. From the image segmented by UNet++ as shown in Fig. 12h, we found that some healthy tissues, which are being detected as lesion. From MultiResUNet, RAUNet, Inception U-Net, and U-Net GAN segmented images in Fig. 12i–l, it has been noticed that MultiResUNet, RAUNet, Inception U-Net and U-Net GAN are properly segmenting the lesions. These four models have achieved decent performance in the detection of lesions. From Fig. 12m,n, it is observed that the performance of V-Net in segmenting the lesion is quite decent, whereas W-Net completely failed to segment the lesion.

A separate comparative study has been conducted with the increased number of MR images having similar characteristics to the dataset used in the first experiment, i.e., a total of 200 MR images is used to test the model. In this experiment, ResUNet achieves $99.23\%$ accuracy, $99.34\%$ precision, $62.10\%$ sensitivity, $72.74\%$ F-measure, $99.65\%$ specificity, $76.08\%$ G-mean, $72.78\%$ DSC, and $0.0035\%$ FPR, which are the best values, and ResUNet retains the first rank when evaluated using TOPSIS.

### Computational complexity

The optimum number of epochs for each model is calculated by hyperparameter tuning using KerasTuner. The computational time of U-Net, ResUNet, Dense UNet, DUNet, Attention U-Net, UNet++, MultiResUNet, RAUNet, Inception U-Net, U-Net GAN, V-Net, and W-Net took are 1406 s, 503 s, 819 s, 502 s, 936 s, 631 s, 1212 s, 1286 s, 2060 s, 326 s, 1025s, and 621s respectively. They all are in the range of 326–1406 s

U-Net GAN runs faster than all the models. Based on Multi-Criteria Decision Analysis, ResUNet is evaluated as the best model with relatively lower training time compared to U-Net. The experimental results revealed that, although models are effective in lesion segmentation, efficiency is an issue that may affect the effectiveness of these models.

## Discussion

In this current study, we investigated in-depth and analyzed the performance of several U-Net models in women’s breast lesion detection using DCE-MRI. Furthermore, the performances of UNet models are also compared with other state-of-the-art deep learning models such as V-Net, W-Net, and DeepLabV3+ models to test their competitiveness. The quantitative results are analyzed using Box-Plots and the non-parametric statistical test method WSRT. Finally, the MCDA method TOPSIS is used to select the best models from thirteen deep learning models and ResUNet achieves the first rank whereas DUNet holds the last rank among the UNet models. The performance of V-Net is superior to DUNet. The ranks of ResNet, U-Net, MultiResUNet, Attention U-Net, Dense UNet, RAUNet, Inception U-Net, U-Net GAN, and UNet++ are higher than that of V-Net, W-Net, and DeepLabV3+. From this observation, it can be concluded that all the U-Net models except DUNet are more effective than V-Net, W-Net, and DeepLabV3+ models in women’s breast lesion detection using DCE-MRI. From the qualitative (visual) results in Figs. 11 and 12, it is also observed that all the U-Net models and V-Net can segment the lesions in breast DCE-MRIs. It is worth noticing that W-Net segments the lesions in the MRI of patient-1 (Fig. 11n) whereas it cannot do the same for the patient-2 (Fig. 12n). DeepLabV3+ is unable to segment the lesions for both patients. Overall, ResNet performs better than all the other models in women’s breast lesion segmentation using DCE-MRI and that observation is supported by both quantitative results analyzed by statistical test WSRT and MCDM ranking, and visual analysis of segmented and localized lesion images. ResUNet is based on the ResNet architecture. The use of skip connections helps to deal with the problem of diminishing gradients and allows the training of complex models, which in turn contributes to faster convergence to the solution^70^. The U-Net model works very well with a small number of annotated images^18^. So it can perform well even on a small dataset. In the MultiResUNet model, convolutional layers between the encoder and the decoder reduce the semantic gap, and the residual connections make the model easy to train^56^. In our study, these are the three best-performing models. The encoding and decoding units in the case of DUNet are deformable convolutional blocks. Also, DUNet performed well in retinal vessel segmentation, which was trained on a large patch dataset^53^.

Apart from the success of most of the U-Net models, there are also some limitations. From the visual results, it is observed that along with the segmentation of lesions, some healthy tissues are also segmented as lesions that are not expected in breast lesion detection. It is also noticed that some lesions are not segmented. This may have occurred due to overfitting of the models during the training process and the models learn the noise and intensity inhomogeneities (IIH) present in the MRIs. Overfitting occurs due to model complexity and the presence of noise and IIH in MRIs affects the segmentation performance. In this study, the hyper-parameters (e.g., learning rate) are set empirically. The gradient-based optimization algorithm called Adam is used to optimize the network’s parameters and gradient-based algorithms generally get stuck in the local minima of the error surface. The breast MRI segmentation problem is a class imbalance problem where the number of pixels corresponding to healthy tissues is very large compared to that of lesions and lesions have very few representations compared to healthy tissues in the training process. The future works can be as follows: (1) model’s *hyper-parameters* tuning using Random Search technique^64^ to improve training performance, (2) Instead of gradient-based learning algorithm, metaheuristics can be used in training of the models to overcome the local minima and class imbalance problems^71^, (3) training and evaluating the models on heterogeneous datasets because there are various changes in images because of sensors and other elements since the nature of data varies from hardware to hardware, and (4) use of suitable denoising and intensity inhomogeneities correction techniques as the preprocessing steps in the proposed segmentation model to enhance the segmentation performance.

## Data Availability

The data-set generated and/or analysed during the current study are available in the The Cancer Genome Atlas Breast Invasive Carcinoma Collection (TCGA-BRCA) repository, https://wiki.cancerimagingarchive.net/pages/viewpage.action?pageId=3539225.
